# Effects of music exposure during pregnancy on maternal behavior in mother rats

**DOI:** 10.1016/j.heliyon.2022.e10029

**Published:** 2022-08-05

**Authors:** Yurika Takano, Masakazu Umezawa, Natsuko Kubota, Ken Takeda, Shinya Yanagita

**Affiliations:** a Graduate School of Pharmaceutical Sciences, Tokyo University of Science, Noda, Chiba 278-8510, Japan; b Research Institute for Science and Technology, Tokyo University of Science, Noda, Chiba 278-8510, Japan; c Department of Materials Science and Technology, Faculty of Advanced Engineering, Tokyo University of Science, Katsushika, Tokyo 125-8585, Japan; d Faculty of Pharmaceutical Sciences, Sanyo-Onoda City University, Sanyo-Onoda, Yamaguchi 756-0884, Japan; e Faculty of Science and Technology, Tokyo University of Science, Noda, Chiba 278-8510, Japan

**Keywords:** Prenatal music, Maternal behavior, Oxytocinergic system, FosB

## Abstract

Several studies have demonstrated the possibility of positive effects of exposure to music during pregnancy on mental function in humans and animals. Although there remains a core belief in the positive effects of music during pregnancy, the underlying neurobehavioral mechanisms of these effects remain unknown. In this study, we aimed to clarify the relationship between maternal nurturing behavior and the oxytocinergic system to elucidate the effect of music on mental health during pregnancy in an experimental investigation using animal models. Pregnant rats were exposed to Mozart sonatas, and their nurturing behavior after delivery was assessed using behavioral analyses. The neural activities of the oxytocinergic system, which are associated with nurturing behavior, were investigated using FosB immunohistochemistry. Music during pregnancy significantly increased the licking behavior of mothers towards pups, which is representative of positive nurturing behavior. In contrast, this alteration in maternal behavior was shown to have no marked effect on the structure or activity of the oxytocinergic system. This study provided possible evidence that exposure to music during pregnancy had a positive effect on postnatal maternal behavior. The results also suggest that the oxytocinergic system, considered a strong candidate for the neural system that regulates maternal behavior, may not be associated with this behavioral change. Understanding the relationship between other neural systems, physiological responses, and nurturing behaviors will provide a more comprehensive explanation of the mechanisms by which music exposure during pregnancy has a positive effect on mental health.

## Highlights

• Pregnant rats were exposed to Mozart sonatas and their nurturing behavior was investigated.
• Exposure to music during pregnancy has a positive effect on postnatal maternal behavior.
• Maternal behavior had no marked effect on the structure or activity of the oxytocinergic neurons.

## 1 Introduction

Music influences humans' mental [1, 2] and physical [3] health in various ways. In particular, listening to Mozart’s music has been reported to improve cognitive and learning functions. This “Mozart effect” has received much attention [4, 5]. A musical environment during pregnancy increases the responsiveness of the fetus to the mother’s voice as well as the music, as assessed by fetal movement and heart rate [6, 7, 8]. Music in the neonatal period has also been reported to have the potential effect of inhibiting resting metabolism and energy expenditure and promoting growth [9, 10]. Thus, music during pregnancy is considered to exert a positive effect on the physical and mental health of mothers and infants. However, the physiological mechanisms underlying these effects remain unclear. An understanding of the mechanisms is needed to identify appropriate and effective ways for using music during pregnancy. Recently, neurobehavioral studies using animal models have begun to investigate the effects of prenatal music exposure on the psychological state of mothers and the development of their offspring. The results revealed interesting effects of prenatal music exposure on brain function, but only in the context of learning and memory [11, 12, 13, 14, 15] and neurogenesis of the hippocampus in the offspring [16]. On the other hand, it is not fully understood how music during pregnancy affects the mother and whether it changes the interaction between mother and offspring.

Early life environments have been linked to understanding mental and physical health across the life span [17, 18]. Prenatal maternal behavior partially programs the epigenetic states of pups [19, 20, 21] and plays an important role in the development of emotional and social behavior and in the acquisition of moderate stress responsiveness in pups [22]. Maternal behaviors such as pup-rearing behavior in rats are altered by environmental factors during pregnancy. The frequency of maternal behaviors is reduced by stress [23, 24, 25, 26], while it is enhanced in enriched environments [27]. Early life maternal care is also associated with later-life behaviors [28, 29]. Maternal behavior is mediated by oxytocin [30, 31], a neuropeptide synthesized in the paraventricular nucleus (PVN) [32]. The central nucleus of amygdala (CeA) expresses oxytocin receptors and interacts with the reward circuit to motivate maternal behaviors [33, 34, 35]. In addition to the oxytocinergic system, previous studies have demonstrated that the neural activity in the median preoptic area (MPOA), the bed nucleus stria terminal (BNST) of mother rats is essential for expressing pup-rearing nurturing behaviors [34, 36, 37, 38, 39, 40]. If music exposure during pregnancy can alter maternal behaviors, it could influence developing brain functions in infants, and, in turn, lead to behavioral changes. Although the relationship between prenatal music exposure and developmental changes has been elucidated, the effects of prenatal music exposure on brain function and the behavior of dams remain unclear.

Thus, we hypothesized that prenatal music exposure may also have a positive effect on maternal behavior. The frequency of maternal pup-rearing behavior, which is essential for the growth of mammalian pups, is known to influence developmental changes in emotional functioning, such as changes in anxiety-like behavior [41], stress responses [42, 43, 44, 45], and cognitive functions [46, 47]. This study sought to investigate the relationship between maternal nurturing behavior and the oxytocinergic system for elucidating the effect of music on mental health during pregnancy by conducting an experimental investigation using rat dams. As a first step towards elucidating the effects of music on mother-pup interaction, the present study focused on the effects of music on maternal behavior during pregnancy.

## 2 Materials and methods

### 2.1 Animals housing and handling

Pregnant female Wistar rats (gestational day 3), purchased from Japan SLC, Inc. (Shizuoka, Japan), were housed separately in a 12-h light/dark cycle at a controlled temperature (23 ± 1 °C), humidity (50 ± 5%) with free access to water and food (Rodent Diet CE-2 for breeding; CLEA Japan, Inc., Tokyo Japan); all experimental procedures using animals were conducted with the approval of the Institutional Animal Care and Use Committee of Tokyo University of Science and related governmental guidelines, which are based on the Animals in Research: Reporting *In Vivo* Experiments. All efforts were made to minimize the number of rats used and their suffering.

### 2.2 Musical exposure for rats during pregnancy

**Figure 1:**
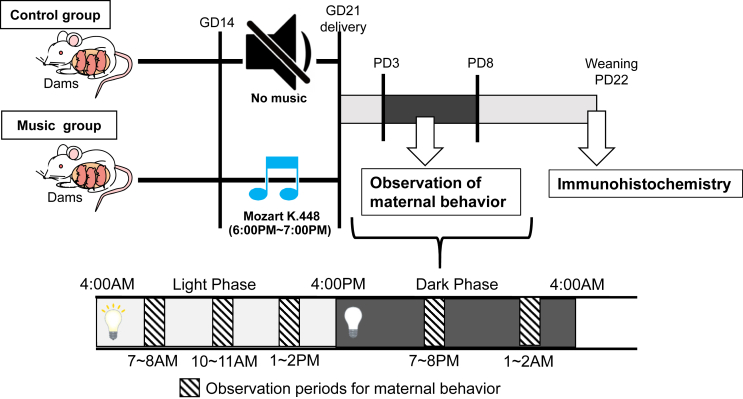
Schematic illustration of the experimental protocol and observation of maternal behavior. Pregnant rats were exposed to Mozart sonatas, and maternal behavior after delivery was assessed using behavioral analyses. Maternal behavior was determined at 3 h of the light and 2 h of the dark periods at postnatal days 3–8. After weaning, brain of the dams were perfused with heparin solution, followed by fixation regent containing 4% paraformaldehyde. The neural activities of the oxytocinergic system, which are associated with nurturing behavior, were investigated using FosB immunohistochemistry. GD, gestation day; PD, postnatal day.

The musical exposure for pregnant rats was conducted in a sound-attenuating room (MC-050; Muromachi Kikai Co., Ltd., Tokyo, Japan) at a sound level of 65 ± 5 dB for 60 min per day, starting at 2 h after lights-out (Figure 1), for 8 days during gestational days 14–21. The rats in the music group were exposed to the “Mozart Sonata for Two Pianos in D major, K. 448,” especially the Andante, which is usually used in studies of the “Mozart effect” [5, 48] while those in the control group were exposed to ambient noise.

The pregnant rats delivered their pups following exposure at gestational day 21. The number of born pups was 3.8 ± 0.6 males and 4.3 ± 0.6 females in the music group, and 4.4 ± 0.7 males and 4.4 ± 0.6 females in the control group (Mean ± SE).

### 2.3 Measurement of pup-rearing behavior and body weight of mother rats

The behaviors of the mother and pup rats were recorded using infrared and visible video cameras to monitor them in both the dark and light periods. The times of licking, contacting, and nursing with an arched-back posture were determined at 3 h in the light and 2 h in dark periods (Figure 1) at postnatal days (PNDs) 3–8, as described previously [29, 49]. The body weight of the mother rats was recorded at gestational days 7, 13, and 17, and PND 21. Brain samples were collected on PND22 instead of PND8, when the analysis of nurturing behavior was completed, to ensure the lactation period, which is essential for child rearing required for analysis of pups.

### 2.4 Acquisition and slice preparation of brain samples

We have previously established immunostaining techniques for the oxytocin nervous system and employed the same procedures and antibodies in this study [50, 51, 52]. Before using oxytocin antibody (ImmunoStar Cat# 20068, RRID: AB_572258) in present study, we tested quality of antibody using standard immunohistochemical methods. This antiserum demonstrated strongly positive labeling of rat hypothalamus compared negative control, in which no labeling cell was detected against non-oxytocin serum proteins, using indirect immunofluorescence and biotin/avidin-HRP techniques. The reagents used in this experiment are listed in Table 1.

**Table 1:** The list of reagents used for brain analysis including immunohistochemistry.

*Purpose for use*	*Reagent name*	*Supplier*
Perfusion and Fixation of Tissue	paraformaldehyde (PFA)	Wako Pure Chemical Industries, Ltd., Osaka, Japan
	glutaraldehyde	Wako Pure Chemical Industries, Ltd., Osaka, Japan
	heparin sodium	Wako Pure Chemical Industries, Ltd., Osaka, Japan
	picric acid	Wako Pure Chemical Industries, Ltd., Osaka, Japan
	sodium azide	Wako Pure Chemical Industries, Ltd., Osaka, Japan
	physiological saline	Otsuka Pharmaceutical Factory, Inc., Tokushima, Japan
Immunohistochemistry	nickel ammonium sulfate hexahydrate	Wako Pure Chemical Industries, Ltd., Osaka, Japan
	phosphate-buffered solution (PBS) (10x, pH 7.4)	Life Technologies Co., Carlsbad, CA, USA
	sodium pentobarbital and Triton X-100	Nacalai Tesque Inc., Kyoto, Japan
	horse serum	Cosmo Bio Co., Ltd., Tokyo, Japan
	tris (hydroxymethyl)-aminomethane hydrochloride	Sigma-Aldrich Co. LLC, St. Louis, MO, USA
	anti-oxytocin antibody (#20068; donor: rabbit)	Immunostar Inc., Husdon, WI, USA
	anti-FosB antibody (SC-48; donor: rabbit)	Santa Cruz Biotechnology, Inc., Dallas, TX, USA
	biotin-conjugated donkey anti-rabbit immunoglobulin G (IgG) (AP182B)	Millipore, Burlington, MA, USA
	avidin/biotin-labeled peroxidase complex (Vectastain Elite ABC Standard kit; PK-6100)	Vector Laboratories, Inc., Burlingame, CA, USA
Microscopic observation	gelatin	Wako Pure Chemical Industries, Ltd., Osaka, Japan
	ethanol	Wako Pure Chemical Industries, Ltd., Osaka, Japan
	xylene	Kanto Chemical Co., Inc., Tokyo, Japan
	Entellan New	Merck KGaA, Darmstadt, Germany

Fixation reagent containing 4% PFA was prepared by mixing 800 mL of 5% PFA aqueous solution, 100 mL of saturated aqueous solution of picric acid, and 100 mL PBS (10x), and stored at 4 °C until use. At PND 22, the mother rats were perfused with physiological saline containing heparin sodium (20 U/L) and thereafter with ice-cold fixative reagent supplemented with 0.1% glutaraldehyde under anesthesia by intraperitoneal injection of sodium pentobarbital (50 mg/kg). The brains were then collected, post-fixed with the fixative reagent without glutaraldehyde for 48 h at 4 °C, replaced in an aqueous solution of 30% sucrose for 3–4 days for cryoprotection, and frozen at −80 °C. To prepare 40-μm-thick slices of the coronal plane, the frozen brains were placed on a sample stage cooled by dried ice and cut using a microtome (Yamato Koki Kogyo K.K., Saitama, Japan). The slices were stored in PBS containing 0.1% sodium azide at 4 °C until further use.

### 2.5 Immunohistochemistry for oxytocin and FosB

Protein expression patterns in the brain slices were analyzed by immunohistochemistry using the free-floating method as described previously [53]. Briefly, the slices were washed with PBS for 5 min (thrice), treated with 0.3% H_2_O_2_ in PBS for 30 min, washed with PBS, and incubated with primary antibody (anti-oxytocin or anti-FosB) diluted (1:500) in PBS containing 10% horse serum and 0.1% Triton X-100 for 18 h. Next, the slices were washed thrice with PBS containing 0.1% Triton X-100 (PBS-TX) for 5 min, and further incubated with biotin-conjugated secondary antibody (anti-rabbit normal IgG) diluted (1:800) by PBS-TX for 2 h. Then, the slices were washed with PBS-TX for 5 min, thrice, and incubated with avidin/biotin-labeled peroxidase complex diluted (1:400) by PBS-TX for 4 h. After washing with PBS-TX thrice, the slices were reacted with peroxidase in 0.02% diaminobenzidine and 0.01% H_2_O_2_ in 0.05 M Tris-HCl (pH 7.6) for 20 min. Immunoreactivity appeared as a brown stain. Reacted slices were finally washed with PBS thrice, placed on gelatin-coated glass slides, dehydrated by treatment with a graded series of ethanol (50%, 75%, 90%, and 100%), a 1:1 mixture of ethanol and xylene, cleared in 100% xylene, and coverslipped with Entellan New mounting medium.

### 2.6 FosB and oxytocin double staining

The brain slices treated, with 0.3% H_2_O_2_, anti-FosB antibody, biotin-conjugated anti-rabbit normal IgG, and avidin/biotin-labeled peroxidase complex, were incubated in a 0.02% diaminobenzidine and 0.01% H_2_O_2_ supplemented with nickel ammonium sulfate in Tris-HCl for 20 min to make the FosB a dark gray-black stain. These slices were then treated with 0.3% H_2_O_2_ for 30 min, washed with PBS, and incubated with anti-oxytocin antibody for 5 days. After washing with PBS-TX for 5 min thrice, they were further treated with biotin-conjugated anti-rabbit normal IgG diluted (1:1000) by PBS-TX for 2 h, washed with PBS-TX for 5 min thrice, incubated with avidin/biotin-labeled peroxidase complex diluted (1:400) with PBS-TX for 4 h, washed thrice with PBS-TX for 5 min, and then reacted with 0.02% diaminobenzidine and 0.01% H_2_O_2_ in 0.05 M Tris-HCl (pH 7.6) without nickel ammonium sulfate for 20 min to make the oxytocin a brown stain. The stained slices were dehydrated, cleared on gelatin-coated glass slides, and coverslipped with mounting medium.

### 2.7 Quantitative analysis of oxytocin- and FosB-positive neurons

Oxytocin- and FosB-positive neurons were counted in the MPOA, BNST, PVN, and CeA [54] of each rat. Two or three slices (80 or 120 μm thick) were subjected to analysis for each target area.

### 2.8 Statistical analyses

All data are shown as mean ± standard error of the mean (SEM). Statistical analyses of the differences in the means of each group were performed by a repeated two-way analysis of variance (ANOVA) with music and time as factors, a post hoc multiple comparison Bonferroni’s test for all behavioral time-series data (control group, n = 9; music group, n = 10), and an unpaired t test for cell count data of immunohistochemistry (control group, n = 5; music group, n = 5). For body weight of the mother rats, we performed an unpaid t test every period (control group, n = 9; music group, n = 10).

## 3 Results

### 3.1 Effects of music exposure during pregnancy on the body weight and pup-rearing behaviors of mother rats

**Figure 2:**
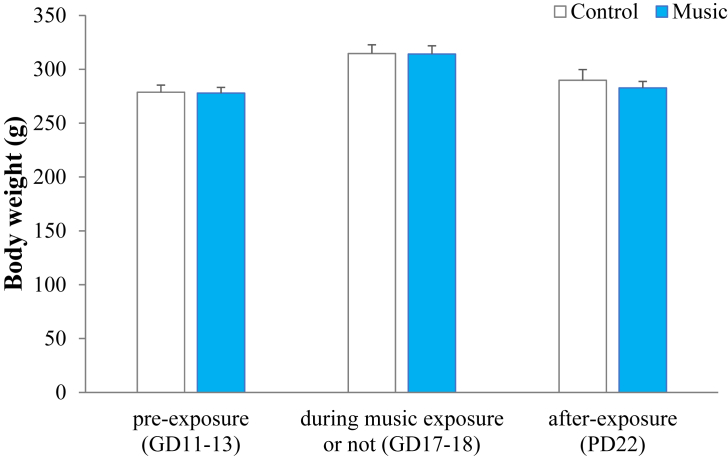
Body weight in dams at pre/during/after-music exposure during pregnancy. There were no significant differences between control and music groups in the body weight of the mother rats at pre/during/after-music exposure during pregnancy. GD, gestation day; PD, postnatal day.

During the gestation (GD17-18) and lactation periods (PD22), there were no significant differences between control and music groups in the body weight of the mother rats (*P* = 0.48; *P* = 0.27, Figure 2).

**Figure 3:**
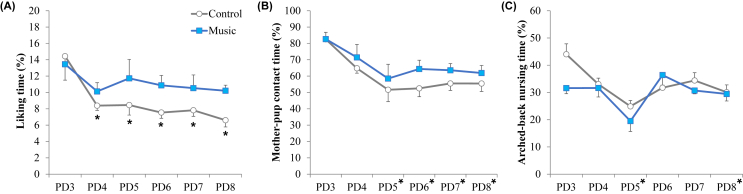
Expression of typical maternal behaviors (mean ± standard error) in mother rats exposed to music or not. Changes in licking time (A), mother–pup contact (B), and arched-back nursing (C) were monitored from the third to the eight day after weaning. (A) Two-way ANOVA showed significant main effects of the day (p < 0.01) and music (p < 0.05) on the liking time without interaction. In control group, mother’s liking time at PDs 4–8 significantly decreased compared to that at PD3. (B–C) Two-way ANOVA showed significant main effects of the day on the mother-pup contact time and arched-back nursing time (p < 0.001, respectively), but no main effects of music and no interaction. ∗p < 0.01, vs. PD3; PD, postnatal day.

The pup-rearing behaviors of the mother rats were monitored from the third to the eighth day post-delivery. A two-way ANOVA demonstrated significant main effects of time (postnatal day) (F [5, 85] = 4.65, *P* < 0.01) and music (F [1, 17] = 4.66, *P* < 0.05) on the licking time without time × music interaction (F [5, 85] = 0.87, *P* = 0.45, Figure 3A). For time-series data in control group, a *post hoc* multiple comparison showed a significant decrease in the mother’s licking time at PDs 4–8 compared to that at PD3 (Figure 3A). In terms of the time of contact, two-way ANOVA showed significant main effect of time (F [5, 85] = 7.01, *P* < 0.001), but no significant main effect of music and no interaction (music, F [1, 17] = 3.79, *P* = 0.07; interaction, F [5, 85] = 0.24, *P* = 0.94, Figure 3B). For time-series data after main ANOVA, the time of contact at PDs 5–8 significantly decreased compared to that at PD3. In terms of the time of nursing with arched-back posture, two-way ANOVA showed significant main effect of time (F [5, 85] = 6.01, *P* < 0.001), but no significant main effect of music and no interaction (music, F [1, 17] = 2.49, *P* = 0.13; interaction, F [5, 85] = 1.76, *P* = 0.13, Figure 3C). For time-series data after main ANOVA, the time of contact at PD5 and PD8 significantly decreased compared to that at PD3.

No significant correlation was found between the number or sex of pups born and licking behavior at PD5 (Number of pups; R = 0.5, Sex of pups; R = 0.6, data not shown).

### 3.2 Effects of music exposure during pregnancy on the number of oxytocin-positive neurons

**Figure 4:**
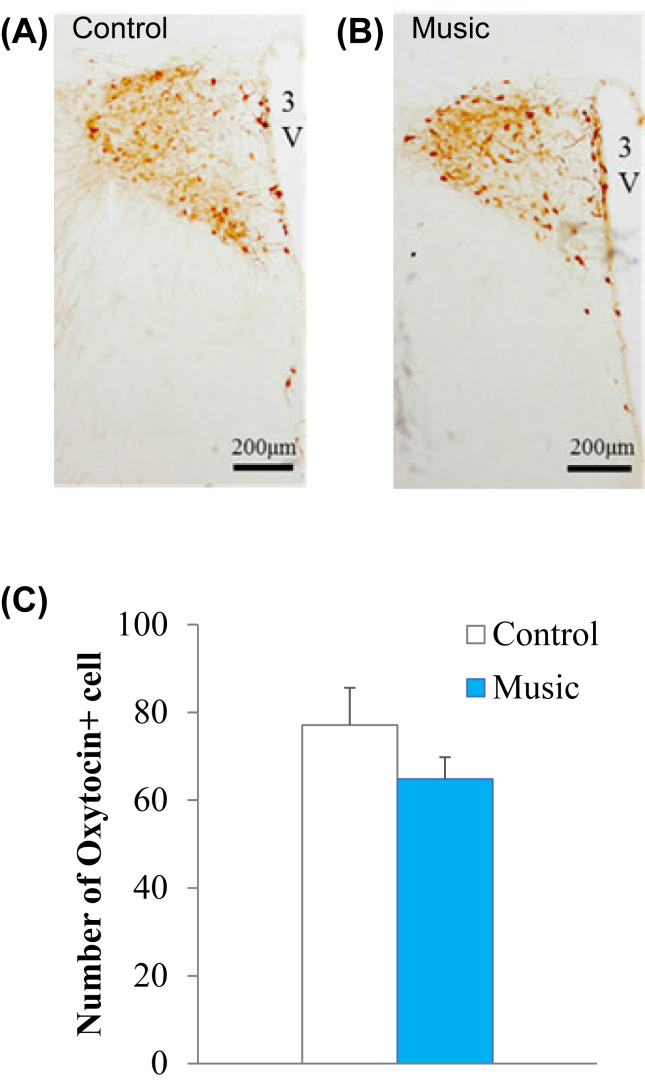
Effects of music exposure during pregnancy on oxytocin neurons in the paraventricular nucleus analyzed by immunohistochemistry. Photographs of oxytocin positive neurons in the paraventricular nucleus (PVN) in control dams (A) and music-exposed dams (B). (C) Number (mean ± standard error) of oxytocin positive neurons in the PVN both conditions. There was no significant difference between the music and control groups of the mother rats at PD22. 3V, third ventricle.

To assess the mechanism underlying the effects of music exposure during pregnancy on the licking behavior of mothers, we examined the number of oxytocin-positive neurons in the mother rats. There was no significant difference between the music and control groups in the number of oxytocin-positive neurons in the PVN of mother rats at PND 22, the day immediately following the lactation period (*P* = 0.12, Figure 4).

### 3.3 Effects of music exposure during pregnancy on neuron activity of target brain areas

**Figure 5:**
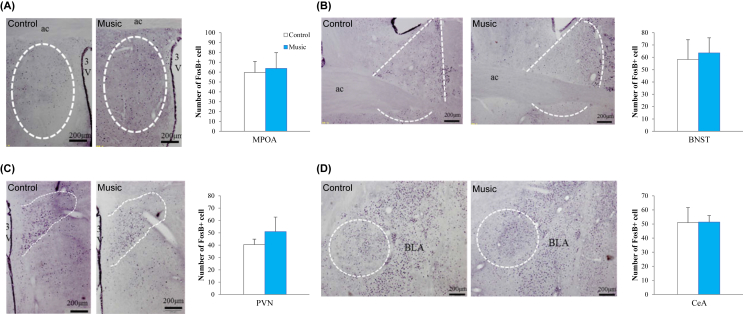
Effects of music exposure during pregnancy on neuronal activation in the brain analyzed by FosB immunohistochemistry. Immunohistochemical images and the average number (mean ± standard error) of FosB immuno-labeled neurons in the brain sections in dams with and without music exposure are shown. The areas in white dashed lines indicate the medial preoptic area (MPOA) (A), bed nucleus of stria terminalis (BNST) (B), paraventricular nucleus (PVN) (C), and central nucleus of amygdala regions (CeA) (D). There were no significant differences between the music and control groups in the MPOA, BNST, PVN, and CeA of mother rats at PD22. ac, anterior commissure; 3V, third ventricle; BLA, basolateral amygdala.

The neuronal activity of brain areas related to the regulation of maternal behavior was investigated by counting the number of FosB-positive cells in each area. There was no significant difference between the music and control groups in the number of FosB-positive neurons in the MPOA (*P* = 0.43, Figure 5A), BNST (*P* = 0.27, Figure 5B), PVN (*P* = 0.20, Figure 5C), or CeA (*P* = 0.49, Figure 5D) of the mother rats at PND 22.

### 3.4 Effects of music exposure during pregnancy on oxytocinergic neuron activity

**Figure 6:**
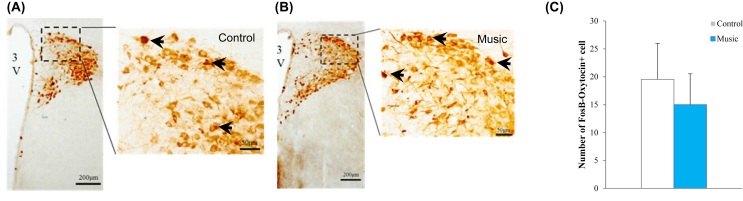
Effects of music exposure during pregnancy on neuronal activation of oxytocin neurons in the paraventricular nucleus (PVN) analyzed by FosB/oxytocin immunohistochemistry. Double-immunolabeled neurons in the PVN of control dams (A) and music-exposed dams (B). Black arrows indicate double-labeled neurons in both FosB and oxytocin. (C) Number (mean ± standard error) of double labeled neurons for FosB and oxytocin in the PVN both conditions. There was no significant difference between the music and control groups of the mother rats at PD22. 3V, third ventricle.

To assess the difference in the oxytocinergic neuron activities related to the regulation of pup-rearing behavior, a quantitative analysis was conducted for the count data on oxytocin–FosB double-positive cells. No significant difference between the music and control groups was identified in the number of oxytocin–FosB double-positive neurons in the PVN of mother rats at PND 22 (*P* = 0.30, Figure 6).

## 4 Discussion

In this study, we aimed to clarify the relationship between maternal nurturing behavior and the oxytocinergic system to elucidate the effect of music on mental health during pregnancy in an experimental investigation using animal models. The results of this study indicate that music during pregnancy significantly increased the licking behavior of mothers towards pups, which is representative of positive nurturing behavior. In contrast, this alteration in maternal behavior was shown to have no marked effect on the structure or activity of the oxytocinergic system.

We found that the “Mozart effect” influenced the licking of offspring by mother rats, a major maternal pup-rearing behavior. In contrast, no significant effect of music was observed on the number of neurons expressing oxytocin, a major factor regulating maternal behavior [30, 31, 40], in the brains of the mother rats. These results suggest that music exposure during pregnancy partially enhances pup-rearing behaviors independent of change in the number or activity of oxytocinergic neurons. Offspring nurtured by mothers with high levels of pup-licking behavior demonstrated reduced behavioral anxiety and stress responses as adults [41, 55]. The results of the present study, which showed that music during pregnancy sustained high frequency pup-licking behavior, suggest positive psychological development in the offspring.

The changes in rearing behaviors due to music are likely caused by the modulation of the oxytocinergic system, such as activation of oxytocin receptors and neuromodulation of the oxytocinergic system via other neuropeptides. Dams who engaged in more pup-rearing behaviors showed expression changes in oxytocin receptors [33, 34, 36, 56]. Contrary to the results of these previous studies, in this study, no significant effect was observed by music during pregnancy on the number of oxytocin neurons and long-term neural activity in the hypothalamus. The reasons for this discrepancy are not clear at this point, but there are several possibilities. The oxytocinergic system receives a wide range of neural inputs and has been shown to be modulated by several other neuropeptides in the regulation of social and emotional behavior, including maternal behavior. For instance, maternal behaviors, including licking, are influenced by dopaminergic nervous system activity [57, 58], which regulates the oxytocinergic system [59]. Arginine vasopressin co-localizes with oxytocin in the hypothalamus and plays an important role in the regulation of maternal behavior [52]. The results from these studies suggest that the oxytocinergic system regulates maternal behavior by receiving diverse modulations from other neuropeptides and non-peptides. Due to the homeostatic stability of these modulations, the number of oxytocin neurons and associated neural activity may not have been affected, as shown in the results of this study. In fact, there are a remarkable number of studies indicating that severe stress during pregnancy reduces maternal nurturing behaviors and oxytocin receptor activity, although very few studies have shown that positive stimulation promotes these processes [23, 24, 25, 26]. Therefore, it is suggested that there may be a homeostatic regulatory mechanism for changes in social and emotional behavior involving the oxytocinergic system; which is supported by the results of previous studies demonstrating only changes in oxytocin receptors due to maternal behavior [30, 31]. Several human studies examined the effects of music in pregnancy [60, 61, 62, 63, 64, 65, 66, 67], but very few have attempted to elucidate the underlying mechanisms of these effects using animals. Although no significant effects were observed in the number of neurons or long-term neural activity in the oxytocin nervous system or selected brain regions of focus in the present study, it is possible that plasticity evaluations, such as changes in receptors and second messengers, could explain these results. In the present study, the short-term evaluation after the music interaction was conducted, so it is not possible to adequately account for the responses that occurred in the intra-cellular system. Studies that change the duration of the interaction would provide new insights.

In addition to the oxytocinergic system, the neural activity in the MPOA [35, 37] and BNST [37, 38] is essential for expressing nurturing behaviors. The PVN represents the higher integration center of the neuroendocrine system [39] and the CeA also expresses oxytocin receptors and interacts with the reward circuit to motivate maternal behaviors [35]. Long-lasting neural activity can be probed by observing protein expression patterns of FosB expressed in cells following neural activation and accumulates, due to its long half-life [68, 69, 70], for at least several weeks [71]. Therefore, we assessed the long-lasting neural activity of the MPOA, BNST, PVN, and CeA regions as reflected in the number of FosB-positive cells in the dams. The results demonstrated no effect for prenatal music exposure on FosB levels in these regions. Rauscher et al. [72] who first reported the “Mozart effect” in humans, suggested that the effect of music is temporary and not persistent. Thus, investigating the activity of various brain regions using c-Fos expression, an index of acute neural activity, may provide an alternative perspective.

While the mechanisms underlying the change in maternal behavior by music exposure during pregnancy remain to be elucidated, the nurturing behavior is controlled by the complex nervous system. Future research will reveal in detail potential changes in the expression levels and activity of oxytocin receptors, and activity of the dopaminergic neuron system or neurotransmission of arginine vasopressin that regulates the oxytocin system in the brain of mother rats. In the present study, we conducted an experimental study of the mother-pup interaction that provides the basis for this positive influence on growth, focusing only on behavioral analysis of mothers. Future work will examine how the positive music-induced behavior observed in mothers influences the development of pups.

## 5 Conclusion

The results of this study suggest that music during pregnancy may influence the initial interaction between mother rats and pups, reflecting their emotional states of mother rats. However, in mother rats, no effect of the music during pregnancy was observed in the neuronal activity of the oxytocinergic system and other systems related to maternal behaviors. Although it is unclear whether changes in maternal behavior due to music during pregnancy incur any effect on the emotional behavior of the pups, the experimental model demonstrated in this study is considered suitable for investigating the effects of music exposure on neurobehavioral and emotional mother–pup interactions. Future research using such experimental models is expected to clarify the effects of the music exposure on the interaction mother and offspring.

## Declarations

### Author contribution statement

All authors listed have significantly contributed to the investigation, development and writing of this article.

### Funding statement

This work was partly supported by the Ministry of Education, Culture, Sports, Science and Technology (MEXT) Supported Program for the Strategic Reuter Foundation at Private Universities.

### Data availability statement

Data will be made available on request.

### Declaration of interest’s statement

The authors declare no conflict of interest.

### Additional information

No additional information is available for this paper.
